# The Intra-Venous Injection of Milk

**Published:** 1878-11

**Authors:** 


*The Intra-Venous Injection of Milk as a Substitute for the Transfusion of Blood.* By T. Gaillaird Thomas, M. D., of New York. Publishers—D. Appleton, & Co., of New York.

This essay is illustrated by seven operations, and consists of an interesting account of a process which presents the possibility of saving life, by the introduction of healthy blood forming material into those suffering from sudden loss or gradual depreciation of the vital fluid.

